# Coupling Effect of LDPE Molecular Chain Structure and Additives on the Rheological Behaviors of Cable Insulating Materials

**DOI:** 10.3390/polym15081883

**Published:** 2023-04-14

**Authors:** Jiacai Li, Zhicheng Si, Kai Shang, Yifan Wu, Yang Feng, Shihang Wang, Shengtao Li

**Affiliations:** State Key Laboratory of Electrical Insulation and Power Equipment, Department of Electrical Engineering, Xi’an Jiaotong University, Xi’an 710049, China; jiacaili@stu.xjtu.edu.cn (J.L.); szcheng@stu.xjtu.edu.cn (Z.S.); shangkai@stu.xjtu.edu.cn (K.S.); wangshih@xjtu.edu.cn (S.W.)

**Keywords:** low-density polyethylene, additives, molecular chain structure, rheological behaviors, cable-insulating materials

## Abstract

The rheological behaviors of low-density polyethylene doped with additives (PEDA) determine the dynamic extrusion molding and structure of high-voltage cable insulation. However, the coupling effect of additives and molecular chain structure of LDPE on the rheological behaviors of PEDA is still unclear. Here, for the first time, the rheological behaviors of PEDA under uncross-linked conditions are revealed by experiment and simulation analysis, as well as rheology models. The rheology experiment and molecular simulation results indicate that additives can reduce the shear viscosity of PEDA, but the effect degree of different additives on rheological behaviors is determined by both chemical composition and topological structure. Combined with experiment analysis and the Doi–Edwards model, it demonstrates that the zero-shear viscosity is only determined by LDPE molecular chain structure. Nevertheless, different molecular chain structures of LDPE have different coupling effects with additives on the shear viscosity and non-Newtonian feature. Given this, the rheological behaviors of PEDA are predominant by the molecular chain structure of LDPE and are also affected by additives. This work can provide an important theoretical basis for the optimization and regulation of rheological behaviors of PEDA materials used for high-voltage cable insulation.

## 1. Introduction

Cross-linked polyethylene (XLPE) with excellent mechanical, heat resistance and insulation performance has been extensively used as an insulating material for high-voltage (HV) cables [1,2,3]. XLPE is generally obtained after dynamic extrusion and crosslinking reaction of low-density polyethylene doped with additives (PEDA) [3,4]. During the dynamic extrusion process, the rheological behaviors of PEDA melt determine its dynamic extrusion molding and insulation structure under high temperature and shear action [4,5,6]. Given that PEDA materials are made of low-density polyethylene (LDPE) by introducing crosslinking agents and antioxidants, the rheological behaviors of PEDA melt are more mysterious and complex [3,7]. Therefore, it is extremely important to reveal the rheological behaviors of PEDA melt for improving the extrusion molding and insulation structure of HV cables.

Nowadays, many works have studied the effect of molecular chain structure on the rheological characteristics of LDPE [8,9]. The zero-shear viscosity has been found to follow an exponential dependence on molecular weight, and with the increase in molecular weight, the shear viscosity rises, but the critical shear frequency of shear thinning decreases [10,11]. When the molecular weight distribution of LDPE is higher, the molecular chains with ultra-high molecular weight have the ability to increase the zero-shear viscosity, and the molecular chains with low molecular weight have the ability to decrease the shear viscosity [11,12,13]. Alongside that, there are some similarities in the effects of polydispersity and long chain branching [14,15]. The zero-shear viscosity and non-Newtonian rise with the increase in long chain branching, and the transition region between the zero-shear viscosity and power law region becomes broadened when the backbone molecular weight of LDPE is same [16,17,18]. Thus, the effect of molecular chain structure on the rheological characteristics of LDPE has made good progress.

However, there are few reports about the effect of additives on rheological behaviors of nonpolar polyolefin, and relevant studies on other polar polymers can provide favorable methodologies for that of nonpolar polyolefin [19,20,21,22,23]. For example, a small number of additives can significantly reduce the viscosity and modulus of polylactic acid (PLA): the lower the molecular weight of additives, the more obvious the reduction degree of viscosity [24,25]. Furthermore, additives with an excessive number of branched carbon atoms and high molecular weight have little effect on the viscosity characteristics of polyvinyl chloride (PVC), and the polar group position of small-molecule additives plays a key role in rheological properties [26,27]. These works have also suggested that a small number of additives can greatly affect the rheological characteristics of polymers. However, the effect of additives on the rheological characteristics of PEDA materials and its mechanism are still unclear. In particular, the coupling effect of LDPE molecular chain structure and additives on the rheological behaviors of PEDA materials is seldom reported.

Here, for the first time, the coupling effect of LDPE molecular chain structure and additives on the rheological behaviors of PEDA is fully revealed. The shear viscosity of PEDA and LDPE materials is obtained by dynamic rheological measurements. Combined with the analysis of LDPE molecular chain structure and viscoelastic constitutive equation, the rheological behaviors of PEDA and LDPE are discussed. Moreover, the effect and mechanism of additives on the rheological characteristics are investigated by experiment and simulation methods. Based on the Doi–Edwards model, the coupling effect of LDPE molecular chain structure and additives on the rheological behaviors of PEDA materials is established. This study can provide an important theoretical basis for the optimization and regulation of rheological behaviors of PEDA materials, which contributes to improving its dynamic extrusion molding and insulation quality.

## 2. Materials and Methods

### 2.1. Materials

Five kinds of commercial PEDA materials used for HV cables were purchased from Borealis, Dow and Yanshan, named C1#, C2#, C3#, C4# and C5#. Dicumyl peroxyide (DCP) and antioxidant 1035 (3,5-Bis(1,1-dimethylethyl)-4-hydroxybenzenepropanoic acid thiodi-2,1-ethanediyl ester)) were purchased from Aladdin. Antioxidant 245 (ethylenebis (oxyethylene) bis-(3-(5-tert-butyl-4-hydroxy-m-tolyl)-propionate)), antioxidant 300 (4,4′-Thio-bis(6-tert-butyl-m-methyl phenol) and 330 (1,3,5-trimethyl-2,4,6-tris(3,5-di-t-butyl-4-hydroxybenzyl)-benzen) were purchased from Macklin.

### 2.2. Based Resin Extraction

An appropriate amount of commercial PEDA materials were put evenly on the mold with the thickness of 0.1 mm, and then placed into a plate-vulcanizing machine for a press under 116 °C and 16 MPa for 10 min. After that, the samples were cooled to indoor temperature under 16 MPa. The obtained samples were cut into a square sample with the size of 10 mm × 10 mm and then these square samples were put into a beaker with adequate absolute ethyl alcohol. After that, the above samples were stirred with magnetic force under 65 °C for 8 h, and then cooled to indoor temperature. The samples above were washed with absolute ethyl alcohol five times. The washed samples were put into a vacuum drying oven under 70 °C for 12 h. Finally, five LDPE materials were obtained and named L1#, L2#, L3#, L4# and L5#.

### 2.3. Sample Preparation

LDPEs were melted under 116 °C and 10 rpm in the cavity of a torque rheometer (RM200C, Hapu Electric Technology Co., Ltd., Harbin, China). The antioxidants and DCP were successively added into the cavity when LDPEs were fully melted. Then, the mixture was stirred under 116 °C and 22 rpm for 10 min. Finally, the self-made PEDA materials with DCP and antioxidants were obtained.

In order to discuss the effect of additives on the rheological characteristics, L5# was selected as the base resin, and seven kinds of self-made PEDA materials with different additives were prepared, as shown in Table 1.

Additionally, in order to discuss the coupling effect of LDPE molecular chain structure and additives on the rheological characteristics, 1.8% DCP and 0.2% antioxidant 300 were added into five kinds of LDPE (L1#, L2#, L3#, L4# and L5#) to prepare the corresponding PEDA materials (namely S1#, S2#, S3#, S4# and S5#).

The above PEDA and LDPE materials were put evenly on the mold with a thickness of 1.0 mm and the circular diameter of 25 mm, and then placed into plate a vulcanizing-machine for a press under 116 °C and 16 MPa for 10 min. After that, the samples were cooled to indoor temperature under the pressure of 16 MPa. These samples were used for dynamic rheological measurements and infrared spectroscopy.

### 2.4. Dynamic Rheological Measurements

The shear viscosity was measured by the stress-controlled parallel plate rheometer (MCR302, Anton Paar (Shanghai) Trading Co., Ltd., Austria, Germany). All experiments were performed under isothermal conditions and the testing temperature was set to 120 °C with an accuracy of ±0.1 °C. During the measurement, dry nitrogen was also continuously purged into the cavity to avoid oxidative decomposition. To avoid damage of the molecular structure of chemical gelation, the dynamic testing pattern was selected as a small-amplitude oscillatory shear mode, and the testing strain was constant at 1%. The frequency scanning range was from 0.01 to 100 rad·s^−1^, and the number of the sampling point was 25.

### 2.5. Molecular Chain Structure of LDPE

Gel permeation chromatography (PL-GPC 50, Agilent Technologies co. Ltd., Santa Clara, CA, USA) was used to obtain the molecular chain structure of LDPE by uniting the differential refractive apparatus and multi-angle laser light scattering apparatus. The parameters of the molecular chain structures were obtained, including weight-average molecular weight (Mw), molecular weight distribution (PD) and long chain branching degree (LCB).

### 2.6. Ingredients of PEDA Materials

The ingredients of the samples were tested by using Fourier transform infrared spectroscopy (Nicolet iN10, Thermo Fisher Scientific Inc., Waltham, MA, USA). All spectra were obtained within the wavenumber range 1800–600 cm^−1^ and the resolution was 4 cm^−1^.

## 3. Results

### 3.1. Rheology Analysis of Commercial PEDA and LDPE

The key rheology parameters of PEDA materials include shear viscosity (η*), zero-shear viscosity (*η*_0_) and the non-Newtonian index (*n*). Considering the requirements for the extrusion molding of PEDA, the anticipant values of η* and *n* are lower and *η*_0_ is higher. Regarding polymers, the viscoelastic constitutive equations have been employed to describe the rheological characteristics [28,29], among which, the Cross model [30,31] is usually used to evaluate the rheological characteristics, as given in Equation (1).

(1) $$\eta *=\frac{\eta_{0}}{1+(\lambda \omega {)}^{n}}$$

where *ω* is the shear frequency and *λ* is the relaxation time. The lower the *n* value, the stronger the non-Newtonian behaviors.

**Figure 1:**
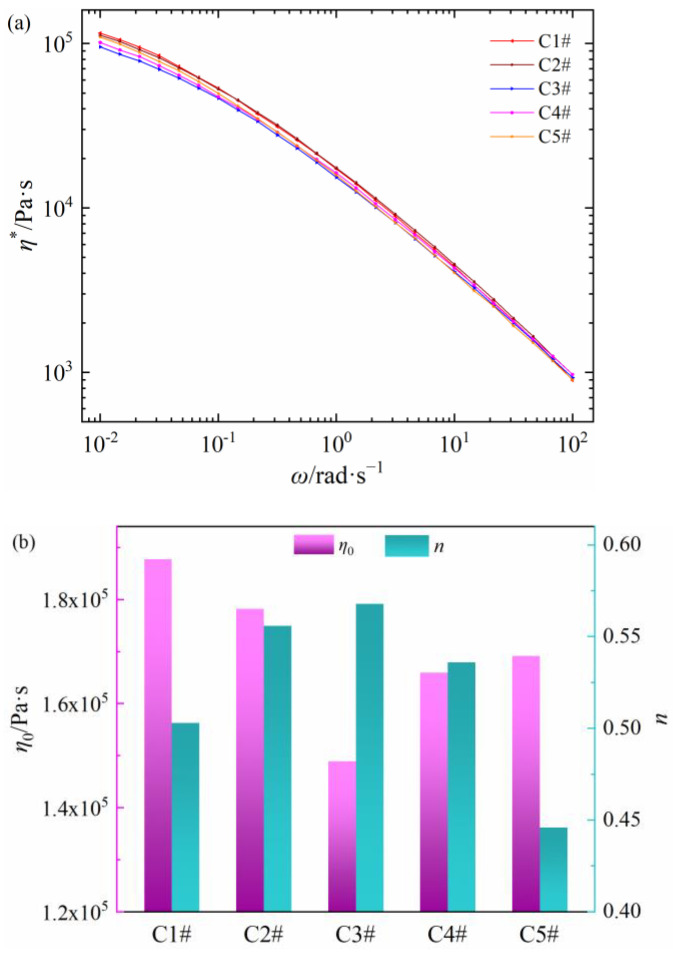
Rheology parameters of different commercial PEDA materials. (**a**) Complex viscosity as a function of shear frequency, (**b**) the zero-shear viscosity and non-Newtonian index.

The relationships between shear viscosity and frequency of five commercial PEDA materials are shown in Figure 1a. All samples present the shear thinning behaviors. With the increase in shear frequency, there is no sufficient time to relax for molecular chains, so that the relative flow resistance between the oriented macromolecules decreases, which leads to the shear thinning behaviors [32,33]. Alongside that, the shear viscosity of five PEDA materials at same shear frequency is different. The η* values of C1# and C2# are higher, while those of C3# and C4# are lower. Additionally, the η* value of C5# decreases quickly with the increase in shear frequency.

The zero-shear viscosity and non-Newtonian index of five PEDA materials are obtained by the Cross model with nonlinear fitting, as shown in Figure 1b. Both *η*_0_ and *n* are different between five PEDA materials. The *η*_0_ value of the five PEDAs is C1# > C2# > C5# > C4# > C3#, and the *n* value is C3# > C4# > C2# > C1# > C5#. The results in Figure 1 indicate that the rheological behaviors of five PEDA materials are quite different. Considering that the PEDA materials are made of LDPE and additives, its rheological behaviors may be determined by both LDPE molecular chain structure and additives.

**Figure 2:**
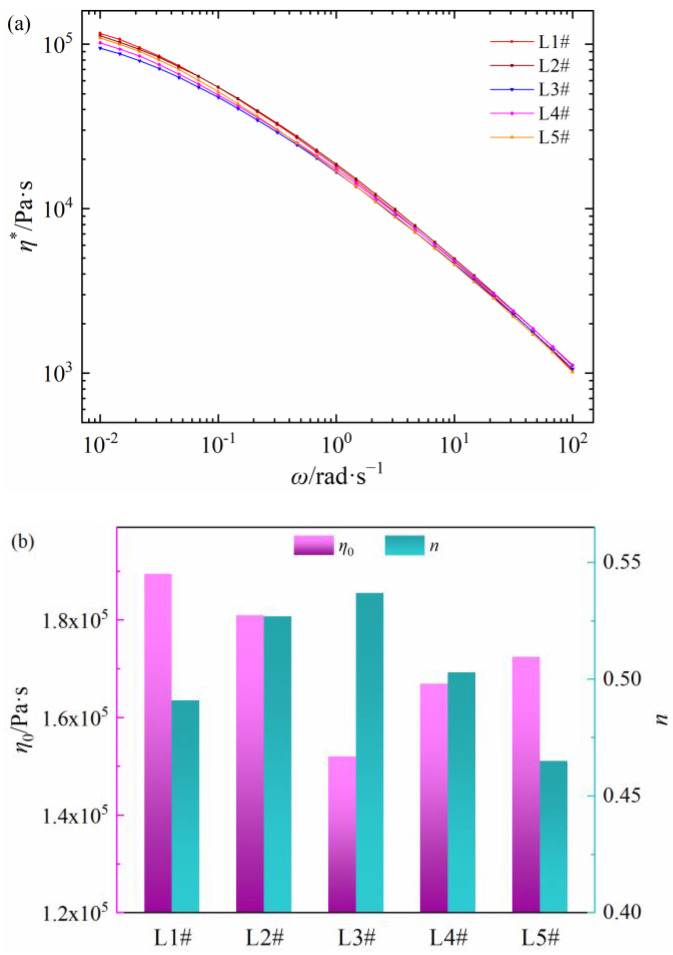
Rheology parameters of different LDPE materials. (**a**) Shear viscosity as a function of shear frequency, (**b**) zero-shear viscosity and non-Newtonian index.

The relationships between shear viscosity and frequency of five LDPEs are shown in Figure 2a. All samples also present the shear thinning behaviors, and the η* values of L1# and L2# are higher, while that of L3# and L4# are lower. Additionally, the zero-shear viscosity and non-Newtonian index of five LDPE materials are shown in Figure 2b. The *η*_0_ value of the five PEDAs is L1# > L2# > L5# > L4# > L3# and the *n* value is L3# > L4# > L2# > L1# > L5#. Compared with Figure 1 and Figure 2, the zero-shear viscosity and non-Newtonian index for the five commercial PEDAs, and their corresponding LDPE are well correlated, respectively. It is worth noting that the zero-shear viscosities for PEDA and LDPE are basically the same, but the non-Newtonian indexes of LDPE have a lower dispersion compared to that of PEDA. Thus, differences in rheological behaviors of commercial PEDA stem from the non-Newtonian indexes, caused by both LDPE molecular chain structure and additives.

These rheological behavior differences of LDPE may be caused by the molecular chain structure. The molecular chain structure, including weight-average molecular weight, molecular weight distribution and long chain branching degree, are collated in Table 2. The *M_w_* and PD values of L1# and L2# are lower, while the LCB is higher, which leads to the higher value of η* and *η*_0_ [11,12,14,16]. The *M_w_* value of L5# is higher and LBC value is lower, which results in obvious non-Newtonian behaviors [11,12,14,16]. Compared with the rheology parameters of PEDA in Figure 1, the *η*_0_ values of corresponding LDPE are basically the same as PEDA, but the η* and *n* values of LDPE are different with corresponding PEDA, which may be caused by additives.

**Figure 3:**
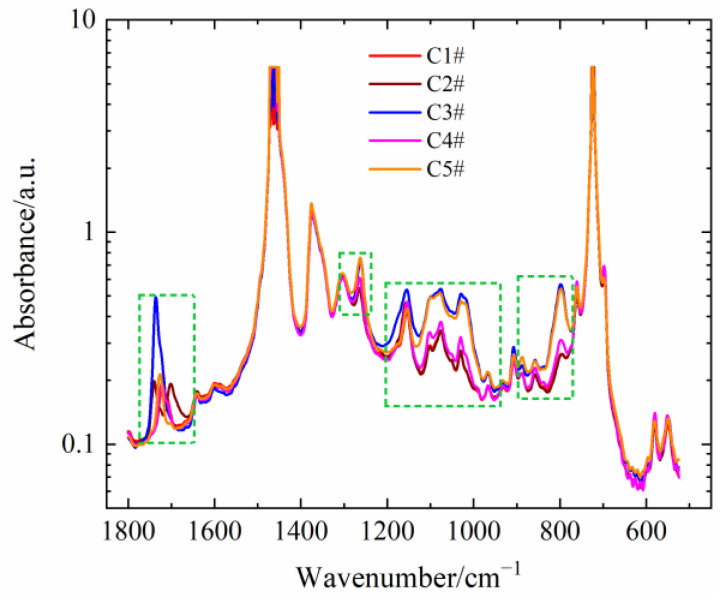
FTIR of different PEDA samples.

The infrared spectroscopy of five PEDA materials is shown in Figure 3, and the absorbance peaks of additives are labeled. As seen in Figure 3, the intensity and position of absorbance peaks are different, which indicates that the kind and content of additives in five PEDA materials are different. Previous works have proven that a small number of additives can greatly affect the rheological characteristics of polymers [24,25,26,27], and thus it is necessary to reveal the effect of different additives on the rheological behaviors of PEDA.

### 3.2. Effect of Additives on the Rheological Behaviors of PEDA

**Figure 4:**
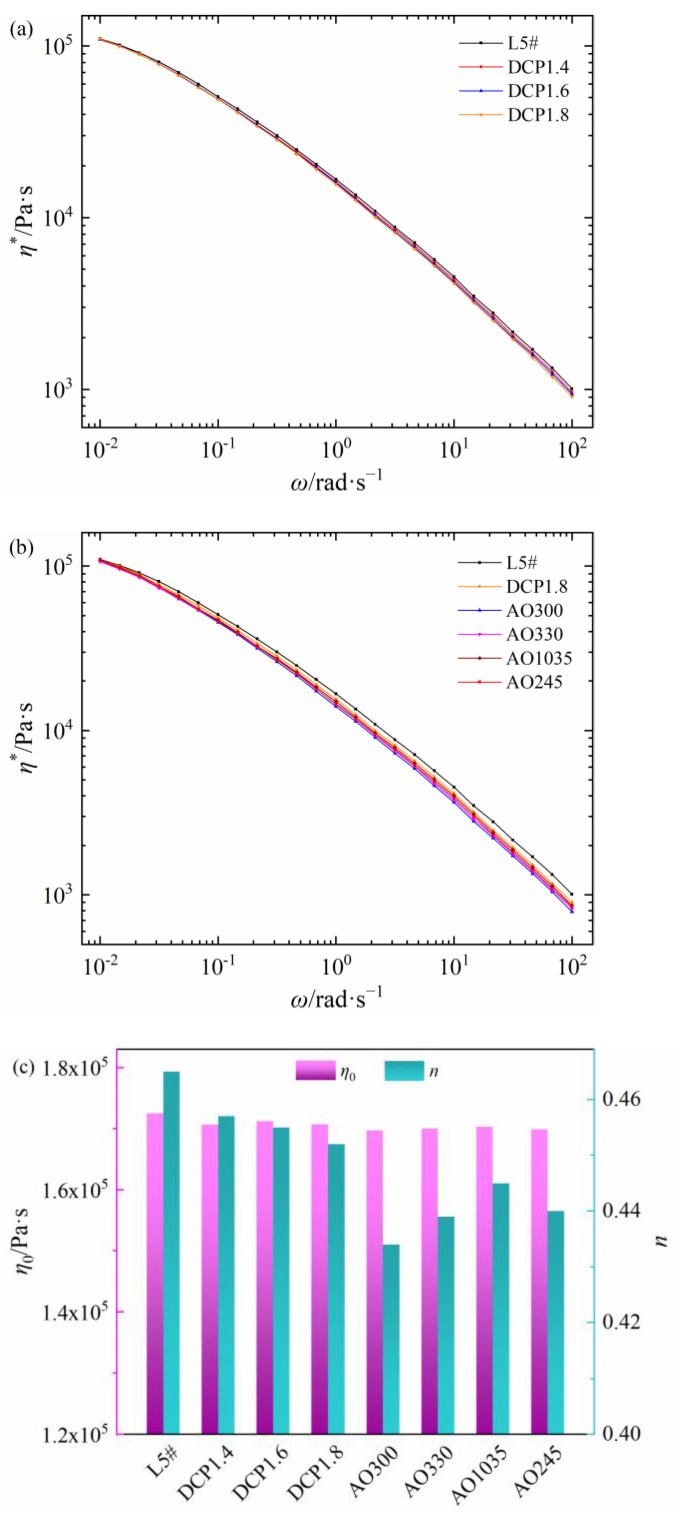
Rheology parameters of self-made PEDA materials. (**a**) Shear viscosity of PEDA with DCP, (**b**) shear viscosity of PEDA with DCP and antioxidants, (**c**) zero-shear viscosity and non-Newtonian index.

The relationships between shear viscosity and frequency of seven self-made PEDA materials are shown in Figure 4a,b. The addition of DCP decreases the η* of PEDA and the η* further decreases with the increase in DCP content. Compared with the DCP, a small amount of antioxidant will obviously reduce the η*, but different antioxidants have different influencing degrees on the η* of PEDA. As seen in Figure 4c, the DCP and antioxidants have little effect on the *η*_0_ of PEDA, but a different influencing degree on the non-Newtonian index of PEDA. Among these, antioxidant 300 has the greatest effect on the rheological behaviors of PEDA, while antioxidant 1035 has the least effect. These results demonstrate that additives can change the rheological behaviors of PEDA materials, but different additives have different influencing degrees.

The influencing degree of additives on the rheological behaviors of polymers is determined by the interaction force and steric hinderance effect. On the one hand, the polar groups of additive molecules interact with macromolecular chains, which weakens the interaction between macromolecular chains, contributing to the relaxation of the macromolecular chains. For another, the steric hindrance effect of additive molecules is larger, and the relaxation of macromolecular chains will be hindered to a certain extent. In order to explain the effect mechanism of additives on the rheological behaviors of PEDA, the chemical composition and topological structure of additive molecules are discussed at the atomic level. The molecular simulation methods and models are presented in Supplementary Materials.

**Figure 5:**
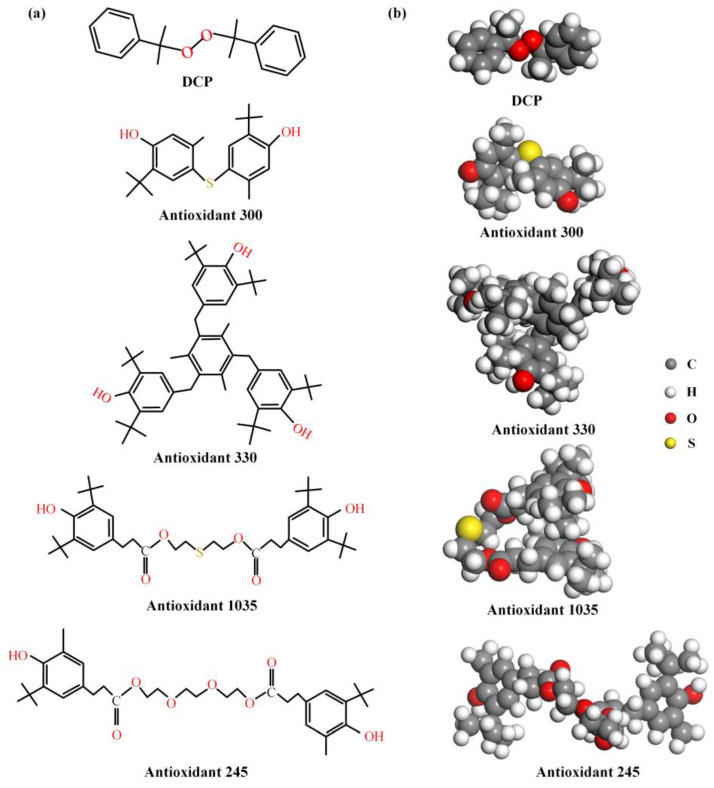
Molecular structure of DCP and antioxidant molecules. (**a**) Planar structure, (**b**) stereochemical structure (extract from the optimized molecular models, as shown in Figure S2 in Supplementary Materials).

The molecular structure of DCP and four antioxidants is quite different, as shown in Figure 5. The molecular weight and number of polar groups can be obtained from Figure 5a, as collated in Table 3. DCP molecules have the least value of both molecular weight and the number within polar groups, but their effect on the rheological behaviors of PEDA are lower than those of antioxidants, which indicates that the effect of the polar group number on the rheological behaviors of PEDA is significantly higher than that of molecular weight.

In addition, the polar group number of antioxidant 300 and antioxidant 330 molecules is the same, but the effect of antioxidant 300 on rheological behaviors is higher than that of antioxidant 330, which means that a higher molecular weight will weaken the effect of antioxidants on the rheological behaviors when the number of polar groups is the same. However, antioxidant 1035 has more polar groups and a lower molecular weight than antioxidant 330, but its effect on rheological behaviors is the lowest, which shows that there are other molecular structure factors of antioxidants in affecting the rheological behaviors of PEDA. The comparison of antioxidant 330 and antioxidant 245 also supports this opinion.

The stereochemical structure of DCP and antioxidant molecules extracted from the optimized models are shown in Figure 5b. The DCP molecule is high in symmetry, and its oxygen–oxygen bond is located in the middle of the molecule, so that its interaction with the LDPE molecular chain is weaker, which explains that DCP has a lower influence on rheological behaviors of PEDA. Moreover, there are three polar groups that are located on the molecular surface in antioxidant 300 molecule, which is eases the formation of non-bond interactions with LDPE molecular chains, and thus the interaction force is strong. However, antioxidant 1035 and 245 molecules have two ester groups, of which the polarity is weaker than that of the hydroxyl group [34,35], and the ester group is not fully exposed on the molecular surface, which leads to the weaker interaction between the additives and LDPE.

The above results demonstrate that these differences in the influencing degree cannot be explained simply by the polar group number and molecular weight of additives, while the size and position of polar groups should be considered, namely the effective polar groups. The number of the effective polar group of antioxidant 1035 is similar to that of antioxidant 330, and that of antioxidant 245 is smaller than that of antioxidant 1035. However, the effect of antioxidant 1035 on rheological behaviors of PEDA is weaker than that of antioxidants 330 and 245. Overall, the number of effective polar groups and molecular weight cannot fully explain the effect mechanism of additives on the rheological behaviors of PEDA.

Considering the steric hindrance effect of small molecules, the topological structure parameters of DCP and antioxidant molecules are further calculated, including molecular volume, radius and sphere index [35,36,37,38]. Van der Waals Volume is used to characterize the molecular volume by MS-Atom Volume-Surfaces and the radius of rotation (*R*_g_) represents the radius of small molecules, as given in Equation (2):

(2) $$R_{g}=\sqrt{{\langle {\left\|x_{i}-x_{0}\right\|}^{2}\rangle }_{i=1\ldots n}},$$

where *x_i_* is the mass center of an atom in the molecule and *x*_0_ is the mass center of the molecule.

The sphere index (*S*_I_) is calculated by a geometric relation, as given in Equation (3):

(3) $$S_{I}=\frac{\sum_{i}m_{i}r_{i,\max}^{2}}{\sum_{i}m_{i}r_{i,\min}^{2}}=\frac{mr_{\max}^{2}}{mr_{\min}^{2}},$$

where *m* is the relative mass of the molecule, *m*_i_ is the relative mass of atom *i*, and *r*_max_ and *r*_min_ are the largest and smallest distance from the molecular mass center to the coordinate axis, respectively. *r_i_*_,max_ and *r_i_*_,min_ are the largest and smallest distance of atom *i* from the coordinate axis, respectively.

The topological structure parameters of DCP and antioxidant molecules are collated in Table 4. The molecular shape is an important factor to determine the steric hindrance effect of additive molecules. The molecular radius is smaller and the sphere index is larger, indicating that the molecular shape tends to be linear and oblate, and the steric hindrance effect is lower [37,38]. The shape of DCP is the most linear oblate, and its molecular weight and molecular volume are small, but its effect on the rheological behaviors is obviously weaker than that of the antioxidants, which further proves that the polar groups play a predominant role in affecting the rheological behaviors of PEDA.

Furthermore, the molecular volume is positively correlated with the molecular weight. The molecular volume of antioxidant 300 is smaller and the steric hindrance effect is lower. The molecular volume of antioxidant 330 is slightly larger than that of antioxidant 1035, but much larger than that of antioxidant 300; meanwhile, the smaller molecular radius and larger spherical index of antioxidant 300 mean that it tends to be linear oblate and have a smaller steric hindrance effect, which further explains that antioxidant 300 has a greater effect on rheological behaviors of PEDA.

The larger molecular radius and smaller sphere index of antioxidant 1035 indicate that it tends to be spherical, and its higher steric hindrance effect will hinder the relaxation motion of LDPE molecular chain to a certain extent. Given this, the effect of antioxidant 1035 on the rheological behaviors is obviously weaker than that of antioxidant 300. Additionally, the number of effective polar groups in antioxidant 330 is similar to antioxidant 1035, and the molecular volume of antioxidant 330 is larger than that of antioxidant 1035, but antioxidant 330 tends to be more linear oblate than antioxidant 1035. These topological structure parameters indicate that the effect of the molecular shape of additives on rheological behaviors of PEDA is greater than molecular weight and molecular volume when the number of effective polar groups is similar.

Although the shape parameters of antioxidant 300 are slightly better than those of antioxidant 330, the effect of antioxidant 300 on rheological behaviors is significantly greater than that of antioxidant 330, indicating that the effect of molecular weight and molecular volume on the rheological behaviors is slightly obvious when the number of effective polar groups and shape parameters are similar. This opinion is supported by comparing the results of antioxidant 245 and antioxidant 1035.

To sum up, the effective polar groups of additives is the dominant factor for the effect degree of additives on the rheological behaviors, but not the only factor. Furthermore, the molecular shape of additives is also an important factor for the effect degree of additive on rheological behaviors. Moreover, the influence of molecular weight and molecular volume on rheological behaviors of PEDA are obvious when the number of the effective polar group and shape parameters are similar. Hence, it is necessary to comprehensively consider the chemical composition and topological structure of additive molecules for discussing the effect mechanism of additives on the rheological behaviors of PEDA.

### 3.3. Coupling Effect of LDPE Molecular Chain Structure and Additives on the Rheological Behaviors of PEDA

**Figure 6:**
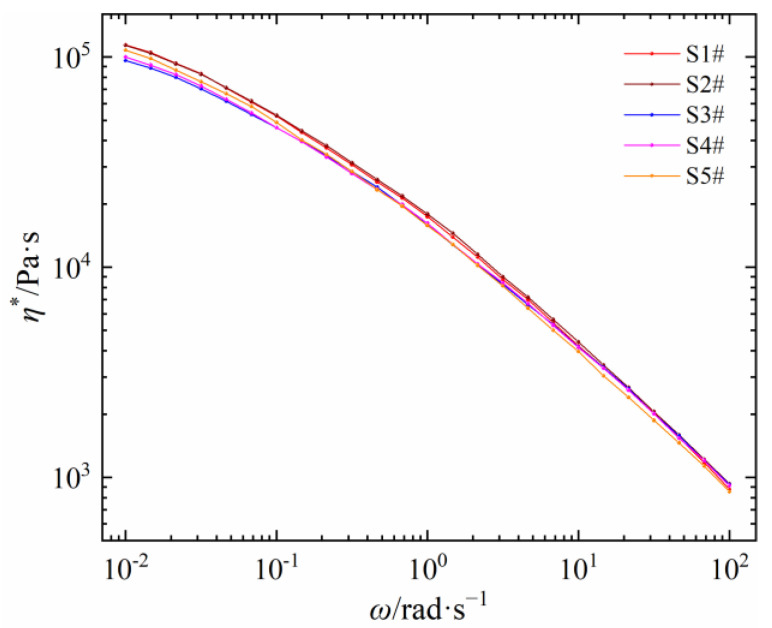
Shear viscosity as a function of shear frequency of different self-made PEDA materials.

Five self-made PEDAs are severally prepared by introducing the same content of DCP and antioxidant 300 into five corresponding LDPEs. The relationships between shear viscosity and frequency of five self-made PEDAs are shown in Figure 6. Compared with the shear viscosity of LDPE in Figure 2a, there are some differences between PEDA and its corresponding LDPE. In the range of shear frequency, the η* values of five PEDAs are lower than that of the corresponding LDPEs, but the decrease degree of five materials is different, which may be caused by the coupling effect of the LDPE molecular chain structure and additives.

**Figure 7:**
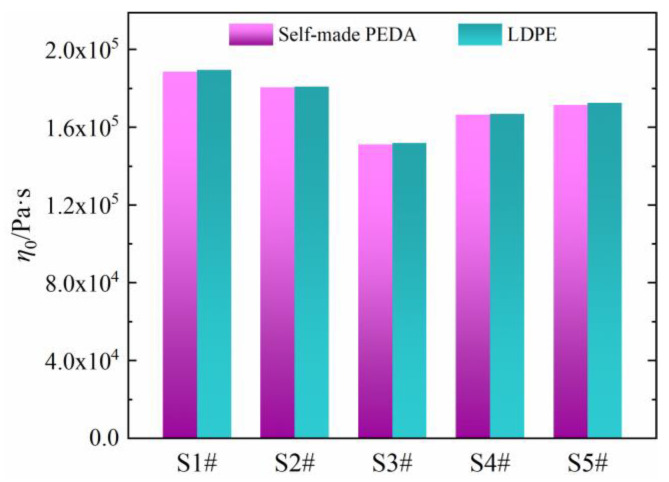
Comparison of zero-shear viscosity between self-made PEDAs and LDPEs.

As seen in Figure 7, the *η*_0_ values are basically the same between PEDA and its corresponding LDPE, which is consistent with the results in Section 3.1, indicating that the *η*_0_ of PEDA is determined only by the molecular chain structure of LDPE, and is not influenced by additives.

In order to quantificationally characterize the differences in rheological behaviors between LDPE and PEDA, their variation degree is calculated and labeled in Table 5. The shear viscosity at the frequencies of 1 rad·s^−1^ and 100 rad·s^−1^ were, respectively, extracted. The decrease degree of η* value is different between five PEDAs compared with their corresponding LDPE. Among these, the η* values of S1# and S2# decrease more than those of S4# and S5#. In particular, the η* of PEDA decreases more at a high shear frequency than that at a low shear frequency. These results fully suggest that the shear viscosity of PEDA is determined by the coupling effect of LDPE molecular chain structure and additives.

Compared with the shear viscosity of 1#, 2# and 3# in Figure 6 and Table 5, the decrease degree in shear viscosity of 3# is bigger than that of 1# and 2#, while the long chain branched degree of 3# is lower than that of 1# and 2#, indicating that the coupling effect between the long chain branched degree and the additives on the shear viscosity is lower. Moreover, the decrease degree in shear viscosity of 4# is bigger than that of 2# and 3#, and the molecular weight of 4# is also higher, which means that the coupling effect between the molecular weight and the additives on the shear viscosity is strong. Furthermore, the decrease degree of shear viscosity of 5# is bigger those that of 3# and 4#, and the molecular weight of 5# is higher than that of 3#, along with the weight distribution of 5# also being higher than 4#, suggesting that the coupling effect between the molecular weight distribution and the additives on the shear viscosity is more obvious.

The non-Newtonian index is another important parameter to describe the rheological behaviors of macromolecules, and the smaller *n* value means that the non-Newtonian behavior is more obvious [13,14]. As seen in Table 5, the *n* values of S1# and S2# increase compared with those of their corresponding LDPE, while the *n* values of S3# and S5# decrease. Combined with the molecular chain structure of LDPE in Table 1, the coupling effect of LDPE molecular chain structure and additives can slightly change the non-Newtonian behaviors of PEDA. It is worth noting that the coupling effect of the molecular weight and its distribution with additives can enhance the non-Newtonian behavior of PEDA when the molecular weight and its distribution is higher. However, the coupling effect between the long chain branching degree and additive can slightly weaken the non-Newtonian behavior.

**Figure 8:**
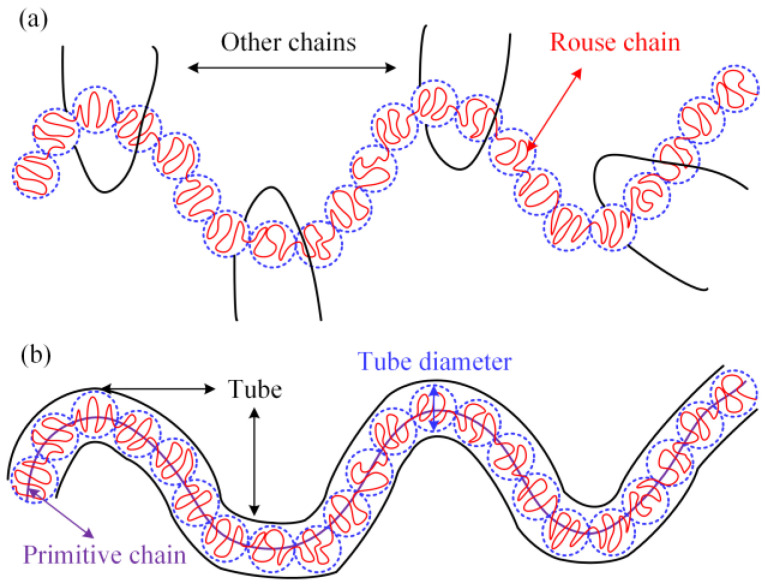
Schematic pictures of the tube model. (**a**) Complex interaction between many chains. (**b**) An effective tube model for a single chain.

In order to explain the mechanism of the effect between LDPE molecular chain structure and additives on the rheological behaviors of PEDA materials, the Doi–Edwards model [39,40] is employed to describe the entanglement characteristics of these systems, as shown in Figure 8.

According to the Doi–Edwards model, the shear viscosity equation can be obtained, as given by Equation (4).

(4) $$\eta \left(\overset{\cdot }{\gamma }\right)=\frac{\sigma \left(t\right)}{\overset{\cdot }{\gamma }}=\frac{G_{e}}{\overset{\cdot }{\gamma }}\int_{0}^{\infty }\mu \left(t\right)\cdot Q\left(\overset{\cdot }{\gamma }\cdot t\right)dt,$$

where ***σ***(t) is the deviatoric stress tensor. *G*_e_ is the relaxation modulus, *μ*(t) is the memory function and ***Q*** is deformation history tensor.

**Figure 9:**
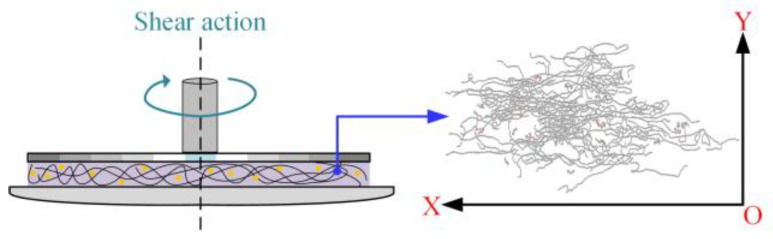
Schematic pictures of the shear flow field in the X–Y plane.

Because the stress-controlled parallel plate and small-amplitude oscillatory shear modes are selected in this measurement, the shear flow field has shear deformation in the X–Y plane, as shown in Figure 9.

Thus, the deformation history tensor equation can be described by Equation (5).

(5) $$Q_{\mathrm{xy}}=\frac{1}{2\overset{\cdot }{\gamma }\cdot t}\int_{0}^{1}\left(1+\frac{x^{2}\overset{\cdot }{\gamma^{2}}t^{2}-1}{\sqrt{x^{4}\left(\overset{\cdot }{\gamma^{4}}t^{4}+4\overset{\cdot }{\gamma^{2}}t^{2}\right)-2\overset{\cdot }{\gamma^{2}}t^{2}x^{2}+1}}\right)dx$$

The zero-shear viscosity can be considered the viscosity value of melt when the shear frequency approaches zero [10,11], and the zero-shear viscosity equation is simplified, as shown in Equation (6):

(6) $$\eta_{0}=\frac{\pi^{2}}{60}G_{e}\tau_{d}==\frac{\chi_{0}cL^{3}}{20aN_{e}^{2}},$$

where *τ_d_* is the relation time, *χ*_0_ is a constant for the friction coefficient of molecular chain, *c* is the number of entangled chains per unit volume, *L* is the length of primitive chains, a is the tube diameter and *N_e_* is the length of the chain entanglement.

Entanglement is an intrinsic interaction for macromolecular chains. There is no shear force at zero-shear frequency, which has little effect on the conformation and orientation of macromolecular chains. Thus, the entanglement and unentanglement of macromolecular chains are caused by Brownian motion [41]. The rates of entanglement and unentanglement are dynamically balanced and the relation time of primitive chains is higher. Since the internal action between additive molecules and the molecular chain is relatively stable without the external force, the entanglement density will not be changed. Alongside that, the additive molecules are attached to the larger molecule chain, which will hinder the peristalsis of the molecular chains at the local locations. On the other hand, the interaction between additive molecules and macromolecular chains can weaken the interchain interaction and reduces the entanglement length. According to Equation (6), the additives cannot affect the zero-shear viscosity of PEDA materials. Furthermore, the entanglement concentration, tube diameter and primitive chain length are closely related to the molecular chain structure of LDPE, thus the zero-shear viscosity of PEDA is determined by the LDPE molecular chain structure.

The shear viscosity equation is deduced by combining Equations (4) and (5), as given by Equation (7).

(7) $$\eta \left(\overset{\cdot }{\gamma }\right)=G_{e}\int_{0}^{\infty }\frac{1}{\overset{\cdot }{\gamma^{2}}}\mu \left(\frac{x}{\overset{\cdot }{\gamma }}\right)Q\left(x\right)dx=\frac{6ck^{3/2}T^{3/2}a^{1/2}}{\pi^{1/2}\chi_{0}^{1/2}N_{e}^{1/2}L^{3/2}{\overset{\cdot }{\gamma }}^{3/2}}\int_{0}^{\infty }x^{-1/2}Q\left(x\right)dx$$

As seen from the above equation, there is a negative power relationship between shear viscosity and frequency, *η*∝*γ*^−3/2^, which explains that the shear viscosity decreases with increasing shear frequency.

According to the Stokes–Einstein formula [42,43], the relationship between diffusion coefficient (*D*) and viscosity can be described, as given in Equation (8):

(8) $$D=\frac{k_{B}\cdot T}{\pi \cdot d\cdot R\cdot \eta^{*}},$$

where *R* is the hydrodynamic radius, *T* is the temperature, k*_B_* is the Boltzmann constant and d is a constant which has a value of 4 or 6.

As the shear viscosity decreases, the diffusion movement of the additives increases and the ability of interacting with macromolecular chains enhances, thus reducing the interaction between macromolecular chains. Alongside that, the macromolecular chains are oriented under shear action [44,45], and the tube diameter is reduced. The unentanglement rate and entanglement rate of PEDA materials increase and the entanglement strength decreases, so the additive can reduce the entanglement concentration of macromolecular chains. According to Equation (7), it is concluded that the additives can reduce the shear viscosity of melt, and the higher the shear frequency, the more obvious the reduction degree.

However, different molecular chain structures will cause differences in the primitive chain length, entanglement density and tube diameter, resulting in different effects with additives, which further leads to different degrees of influence on the rheological behaviors of PEDA. Additionally, the polymer melt has instantaneous network structure; that is, the polymer melt can be considered a complex network system, including the complexity of structure and interaction. Here, non-growth evolution model of complex networks is employed to analyze the rheological behaviors of PEDA [46,47], as shown in Equation (9):

(9) $$P\left(h\right)=\frac{p\left(s+1\right)-qs}{p\left(s+1\right)}\cdot {\left[\frac{qs}{p\left(s+1\right)}\right]}^{\left|h-h_{0}\right|},$$

where *P*(*h*) is the distribution probability of node, *p* and *q* are the probability of unentanglement and entanglement, respectively, *s* is the average number of entanglement points and *h* is the degree of the node.

**Figure 10:**
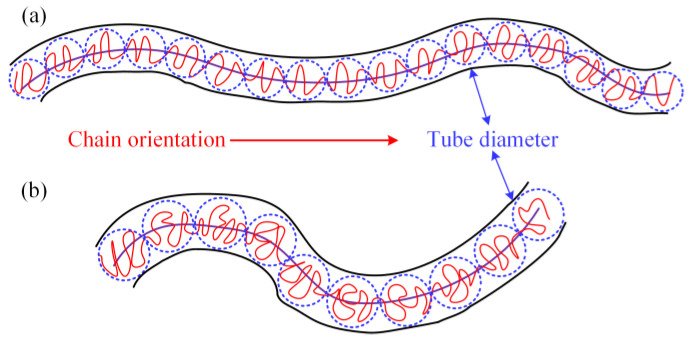
Schematic pictures of the tube model. (**a**) LDPE with a high molecular weight, (**b**) LDPE with a high long branching chain degree.

As shown in Figure 10a, the LDPE molecular chain with a higher molecular weight is more prone to orientation, which leads to the decrease in tube diameter. Meanwhile, the length of the primitive chain and entanglement density increase with the increase in LDPE molecular weight. Thus, the probability of the chain segment interacting with the additive molecules is greater. The dynamic process of chain entanglement and unentanglement is more likely to occur at smaller shear frequencies; that is, there is a tendency to enhance the non-Newtonian behaviors of PEDA when the molecular weight of LDPE is higher. In addition, the probability of the chain segment interacting with the additive molecules is greater, indicating that the probability of unentanglement is higher than that of entanglement. According to Equation (9), the distribution probability of the node will obviously decrease [46,47], so the average number of entanglement points reduces, which means that the shear viscosity decreases more. Overall, the additives will obviously decrease the shear viscosity and increase the non-Newtonian behaviors of PEDA when the molecular weight of LDPE is higher. Because the rheological behaviors of LDPE with a higher molecular weight distribution are determined by the ultra-high and the molecular chains with an ultra-low molecular weight, its coupling effect with additives is similar to that between molecular weight with additives.

In Figure 10b, given that the relaxation time of the molecular chain with the branching chain is shorter, the tube diameter is larger with the increase in long branching chains in LDPE molecules, and thus, the entanglement density will reduce. At this time, the probability of LDPE molecular chains interacting with additives will decrease. Since the relaxation motion of the branching chain is easier, it has a smaller effect on the probability of entanglement and unentanglement of the system when the additive interacts with the branching chains. According to Equation (9), the additives can slightly decrease the distribution probability of the node, so the average number of entanglement points reduces less, indicating that the shear viscosity decreases less. Therefore, the coupling effect between the long branching chain degree and additives is weaker than that of molecular weight.

Combined with the Doi–Edwards model and the non-growth evolution model of complex networks, the coupling effect between additives and LDPE molecular chain structure, and its mechanism on the rheological behaviors of PEDA, are comprehensively explained. It is suggested that the rheological behaviors of PEDA are predominant influenced by the molecular chain structure of LDPE and are also affected by additives, whereas different molecular chain structures of LDPE have different coupling effect degrees with additives.

## 4. Conclusions

In this work, for the first time, the coupling effect between additives and LDPE molecular chain structures on the rheological behaviors of PEDA are fully revealed under uncross-linked conditions. The conclusions obtained are described below.

The rheological behaviors of PEDA are predominantly influenced by the molecular chain structures of LDPE and are also affected by additives. Notably, the zero-shear viscosity of PEDA is only determined by the molecular chain structure of LDPE, and the shear viscosity and non-Newtonian feature of PEDA are affected by the coupling effect between additives and molecular chain structures of LDPE. Moreover, the effect degree of different additives on rheological behaviors is determined by both chemical composition and topological structure. Among these, the effective polar groups and molecular shapes of additives play key roles in the effect degree of additives on rheological behaviors of PEDA. Furthermore, the coupling effect between additives and different molecular chain structures of LDPE on the rheological behaviors is quite different; that is, the LDPE with a higher molecular weight and distribution can form more obvious coupling effects with additives on the rheological behaviors of PEDA, including the decrease in shear viscosity and the increase in non-Newtonian feature.

This work can provide an important theoretical basis for the optimization and regulation of rheological behaviors of PEDA materials, contributing to the improvement of the dynamic extrusion molding and insulation quality of high-voltage cables.

## Figures and Tables

**Table 1:** Ingredients of seven self-made PEDA materials based on L5#.

DCP Content	0.2% Antioxidant	Label
1.4%	/	DCP1.4
1.6%	/	DCP1.6
1.8%	/	DCP1.8
1.8%	antioxidant 300	AO300
1.8%	antioxidant 330	AO330
1.8%	antioxidant 1035	AO1035
1.8%	antioxidant 245	AO245

**Table 2:** Molecular chain structure of five LDPE samples.

Sample	*M_w_* (g∙mol^−1^)	PD	LCB
L1#	119,191	5.347	1.57
L2#	130,321	5.238	1.16
L3#	123,333	7.134	0.74
L4#	197,101	7.867	0.77
L5#	199,261	9.706	0.71

**Table 3:** Chemical composition parameters of additive molecules.

Additives	Molecular Weight	Polar Group Number	Effective Polar Group Number
DCP	270.372	1	0
300	358.540	3	3
330	775.215	3	3
1035	642.936	5	3
245	586.750	4	2

**Table 4:** Topological structure parameters of additive molecules.

Additives	Molecular Volume/Å	Molecular Radius/Å	Sphere Index
DCP	294.61	3.72	5.0563
300	378.61	3.92	3.8678
330	831.59	5.69	3.6461
1035	758.28	7.91	1.7754
245	639.11	6.24	2.9330

**Table 5:** Comparison of rheology parameters between self-made PEDA and LDPE.

	η* at 1 rad·s^−1^ (Pa·s)			η* at 100 rad·s^−1^ (Pa·s)			*n*		
	LDPE	PEDA	Radio	LDPE	PEDA	Radio	LDPE	PEDA	Radio
1#	18,100	17,430	−3.84%	1001	896	−11.72%	0.491	0.503	+2.44%
2#	18,620	17,920	−3.76%	1044	942	−10.82%	0.527	0.536	+1.71%
3#	16,670	15,870	−5.06%	1055	927	−13.81%	0.537	0.540	+0.56%
4#	17,330	16,210	−6.90%	1060	918	−15.47%	0.503	0.484	−3.78%
5#	16,470	15,400	−6.94%	1030	881	−16.91%	0.465	0.442	−4.95%

## Data Availability

Not applicable.

## Supplementary Materials

The following supporting information can be downloaded at: https://www.mdpi.com/article/10.3390/polym15081883/s1, Figure S1. Planar models of DCP and antioxidant molecules. Figure S2. Optimized molecular models, (a) LDPE, (b) DCP, (c) AO300, (d) AO330, (e) AO1035, (f) AO245. Figure S3 Molecular chain conformation of cell model, taking AO300 for example. Table S1 Detailed parameters of molecular models. Reference [48] is from the supplementary materials.
