# Supplementary material for: Coupling Effect of LDPE Molecular Chain Structure and Additives on the Rheological Behaviors of Cable Insulating Materials
Source: Polymers (Basel). 2023 Apr 14;15(8):1883. doi: 10.3390/polym15081883 (PMC10145786; doi:10.3390/polym15081883)
Supplement: Supplementary file 1 [file polymers-15-01883-s001.zip › polymers-2301050-supplementary.pdf]

Supplementary Materials

Coupling Effect of LDPE Molecular Chain Structure and Additives on the Rheological Behaviors of Cable Insulating Materials

Jiacai Li, Zhicheng Si, Kai Shang, Yifan Wu, Yang Feng*, Shihang Wang, Shengtao Li*

State Key Laboratory of Electrical Insulation and Power Equipment, Department of Electrical Engineering, Xi'an Jiaotong University, Xi'an 710049, China

*Corresponding author:
E-mail: fengyang@xjtu.edu.cn (Yang Feng), sli@mail.xjtu.edu.cn (Shengtao Li)

1 Simulation methods and models

1.1 Atomic models for additives

Materials Studio software is used to build two LDPE molecular chains. The polymerization degree and branching degrees are 160 and 0.075, respectively. The number of the long-branch chain is 3 and the molecular weight is 13046.66g/mol. Then, small molecule models of DCP and three antioxidants are established, as shown in **Figure S1**.

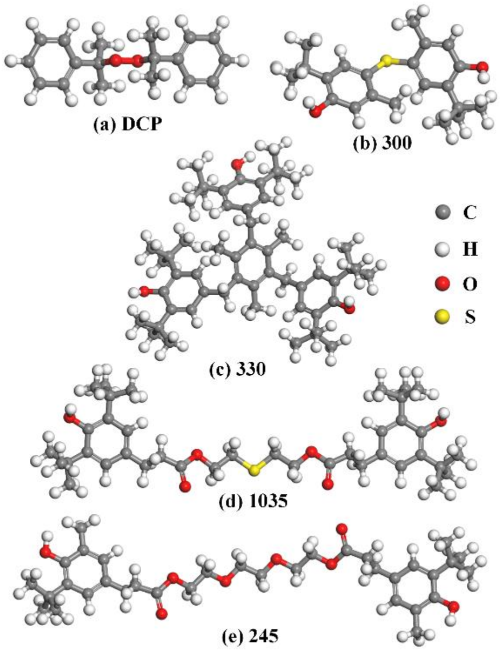

Figure S1. Planar models of DCP and antioxidant molecules.

1.2 Dynamic optimization for molecular models

The Geometry Optimization module is employed to optimize the structure of the two molecular chains, and ten optimized molecular chains are used to construct the periodic amorphous cell models. Amorphous Cell module is used to import LDPE, DCP and antioxidant molecules into periodic cell, in which the mass fraction of antioxidant is the same. The structure optimization and dynamics optimization of the models are carried out as follows.

(1) The Geometry Optimization module is used to optimize the structure of the models until energy stability and structural convergence are satisfied.

(2) The Dynamics module is used to balance the models for 200 ps at 393 K in the canonical ensemble (NVT) so that all the atoms get their initial velocity at this temperature.

(3) The Dynamics module is used to relax the models for 500 ps at 393 K and 20 MPa in the isothermal-isobaric ensemble (NPT) to achieve a stable density.

(4) The relaxed model is annealed from 393 K to 593 K 15 times in NVT and the dynamics process is performed 20 times in each cycle.

(5) Dynamics module is used to perform the equilibrium process of models with the lowest energy after annealing for 500 ps at 393 K in NVT.

In the whole simulation, Compass II force field is selected to calculate. Andersen law is selected for temperature control and Berendsen law is selected for pressure control. Ewald method is selected for electrostatic and Atom based method is selected for van der Waals force. The cutoff distance is 12.5 Å and the calculation step length is 1 fs. The final information of models are shown in **Figure S2** and **Table S1**.

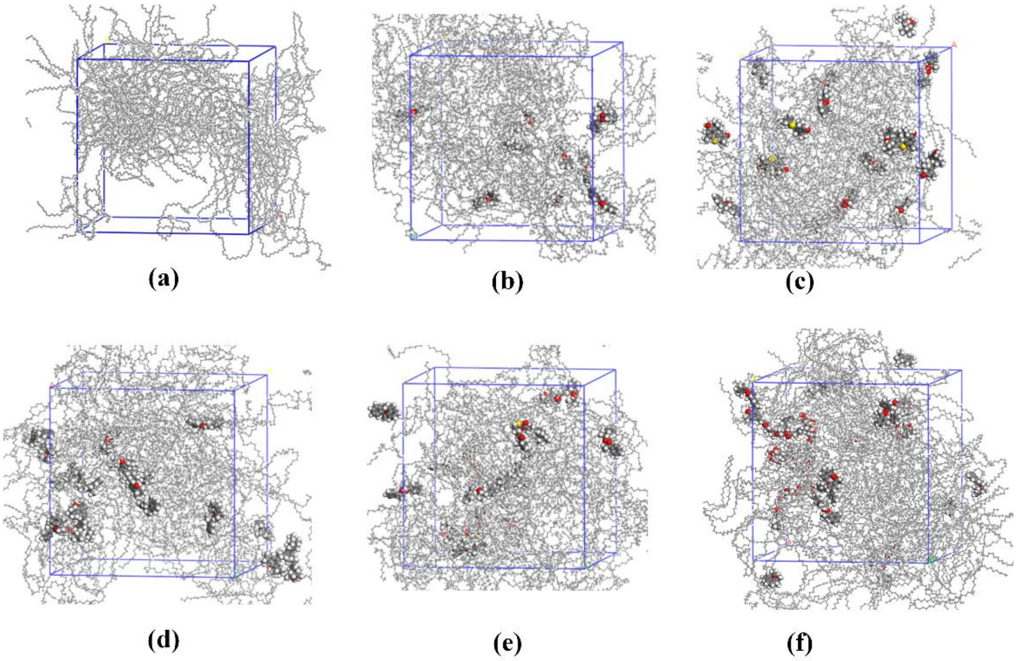

Figure S2. Optimized molecular models, (a) LDPE, (b) DCP, (c) AO300, (d) AO330, (e) AO1035, (f) AO245.

Table S1 Detailed parameters of molecular models

Model	LDPE chain number	DCP number	Antioxidant number	Size /Å	Density/ g·cm^{-3}
LDPE	9	/	/	62.489	0.869
DCP	9	15	/	63.076	0.867
AO300	9	15	7	63.328	0.864
AO330	9	15	3	63.283	0.864
AO1035	9	15	4	63.280	0.866
AO245	9	15	4	63.325	0.865

1.3 Reliability analysis for molecular models

Previous works have proved that the molecular chain will produce an entanglement effect when the polymerization degree of the LDPE molecular chain is greater than 64. Considering the entanglement characteristics and the calculation efficiency of molecular simulation [34, 48], the degree of polymerization of LDPE chains is selected as 160 in this paper. To further evaluate the

validity of the above molecular models, the molecular chain conformation and cell density are analyzed. The cell model with DCP and antioxidant 300 is taken as an example, as shown in Figure S3. The DCP and antioxidant molecules are evenly dispersed, and two molecular chains are arbitrarily selected to meet the characteristics of condensation entanglement and topological entanglement of LDPE molecular chains. In addition, the density of the cell model is shown in Table 1. The simulated temperature is 120°C, and its density close to 0.9 g/cm3 is reliable and reasonable.

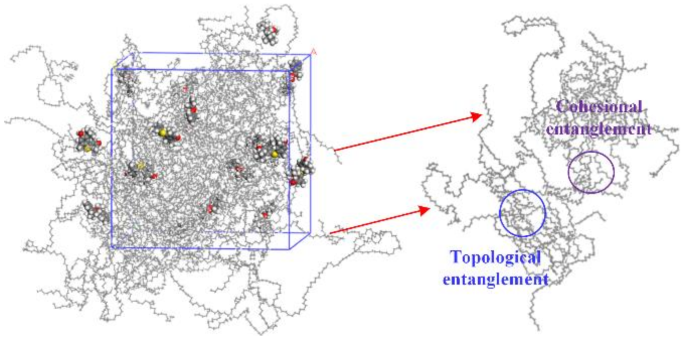

Figure S3 Molecular chain conformation of cell model, taking AO300 for example.

1.4 Methods for shear action

Based on the above models, the MS-Forcite-Shear module is used to simulate the shear conformation of PEDA. The whole calculation process is in the canonical ensemble (NVT) under 120°C for 100 ps. The shear frequency is set as 0.1 ps-1. After that, the LDPE molecular chain conformation of the LDPE molecular chain under the shear action is shown in Figure 9 of the revised manuscript.

References

[34] Sun B.; Lu L.; Zhu Y. Molecular Dynamics Simulation on the Diffusion of Flavor, O_2 and H_2O Molecules in LDPE Film. Materials, 2019, 12, 3515. DOI: 10.3390/ma12213515.

[48] CHO S.; KIM J.M.; BAIG C. Scaling characteristics of rotational dynamics and rheology of linear polymer melts in shear flow. Macromolecules, 2020, 53, 3030-3041. DOI: 10.1021/acs.macromol.9b02184.
